# Supplementary material for: Quantum-enhanced sensing using non-classical spin states of a highly magnetic atom
Source: Nat Commun. 2018 Nov 23;9:4955. doi: 10.1038/s41467-018-07433-1 (PMC6251866; doi:10.1038/s41467-018-07433-1)
Supplement: Supplementary file 1 — Supplementary Information [file 41467_2018_7433_MOESM1_ESM.pdf]

Supplementary information
Quantum-enhanced sensing using non-classical spin states of a highly magnetic atom

Chalopin et al.

Supplementary Note 1: Ideal spin dynamics

We present here the expected dynamics for a pure $\hat{J}_x^2$ coupling. By decomposing the initial state $|-J\rangle_z$ on the x basis $\{|m\rangle_x\}$, we find the evolved state as

$$|\psi(t)\rangle = \frac{1}{2^J}\sum_m \mathrm{e}^{-\mathrm{i}m^2\omega t}(-1)^m\sqrt{\binom{2J}{J+m}}\,|m\rangle_x\,. \quad (1)$$

The magnetization and spin projection variance along z were calculated analytically in Ref. [1], as

$$m_z(t) = -\,J\,[\cos(\omega t)]^{2J-1}\,, \quad (2)$$

$$\Delta J_z^2(t) = J^2\left(1-[\cos(\omega t)]^{2(2J-1)}\right) - \frac{J(J-\frac{1}{2})}{2}\left(1-[\cos(2\omega t)]^{2J-2}\right), \quad (3)$$

corresponding to the dashed red lines in Fig. 2b and c.

The collapse of quantum coherence results from the relative dephasing between the various $m^2\omega t$ phases. For large J values, the evolution of m_z and ΔJ_z^2 are well captured by a gaussian decay on a timescale $t_c = 1/(\sqrt{2J}\omega)$, as

$$m_z^{\mathrm{G}}(t) \simeq -\,J\exp[-(t/t_c)^2/2], \quad (4)$$

$$[\Delta J_z^{\mathrm{G}}(t)]^2 \simeq \frac{J(J+\frac{1}{2})}{2} - [m_z^{\mathrm{G}}(t)]^2 + \frac{J(J+\frac{1}{2})}{2}\exp\left[-\frac{2J^2-2J+1}{J(J-\frac{1}{2})}(t/t_c)^2\right]. \quad (5)$$

We show in the Supplementary Figure 1 the evolution of the magnetization and spin projection variance for $J = 8$. We find that the gaussian approximations of Eqs. (4) and (5) reproduce very well the exact formulas (2) and (3) during the entire collapse ($0 < \omega t < 0.3\pi$).

This gaussian decay of the magnetization and spin projection variance ceases to be valid close to $\omega t = \pi/2$, at which all even (odd) m phase factors get rephased together, leading to a quantum superposition between $|-J\rangle$ and $|J\rangle$ states. More generally, for $\omega t = n\pi/2$ (n integer), one expects the formation of the quantum states

$$|\psi\rangle = \frac{1}{\sqrt{2}}\left(\mathrm{e}^{\mathrm{i}n\pi/4}\,|-J\rangle + \mathrm{e}^{-\mathrm{i}n\pi/4}\,|J\rangle\right), \quad n \text{ odd} \quad (6)$$

$$|\psi\rangle = \left|(-1)^{n/2}J\right\rangle, \quad n \text{ even}. \quad (7)$$

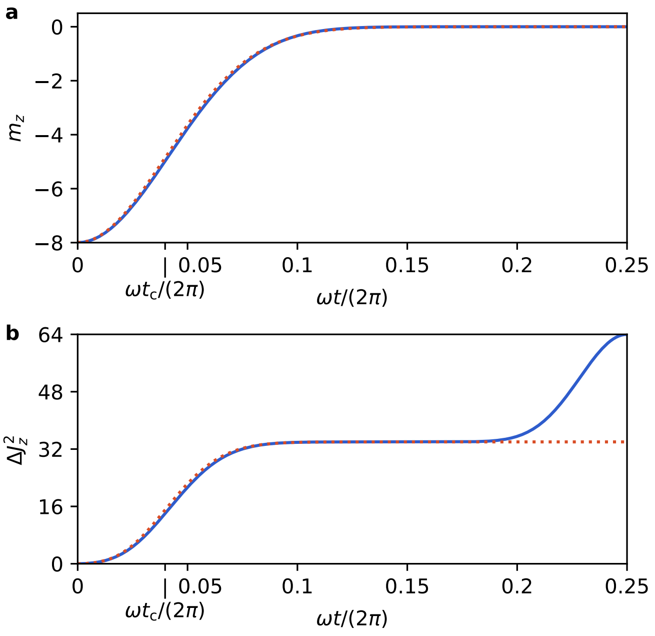

Supplementary Fig. 1. Evolution of the first spin moments during the collapse. (**a**, **b**) Evolution of the magnetization m_z (**a**) and of the spin projection variance ΔJ_z^2 (**b**) under a pure $\hat{J}_x^2$ coupling. The solid blue lines corresponds to the exact expressions (2) and (3). The red dotted lines represent their gaussian approximations given by Eqs. (4) and (5).

Supplementary Note 2: Spin dynamics modeling

The non-linear spin dynamics results from the spin-dependent light shifts caused by laser light whose wavelength is close to the optical transition at $\lambda_0 = 626\,\mathrm{nm}$, that couples the electronic ground state to an excited level of angular momentum $J' = J+1$. The light frequency is detuned from resonance by $\Delta = -2\pi\times 1.5\,\mathrm{GHz}$, a value chosen to maximize the light shift amplitude while keeping incoherent light scattering negligible over the timescale of our experiments. For such a detuning the contribution from other optical resonances to the light shift is negligible.

The light-induced spin coupling, whose structure depends on the light polarization $\hat{\mathbf{u}}$, is obtained using second-order perturbation theory as

$$\hat{V} = V_0\left\{\frac{2J+3}{3(2J+1)}\mathbb{1} - i\frac{2J+3}{2(J+1)(2J+1)}[\hat{\mathbf{u}}^*\times\hat{\mathbf{u}}]\cdot\hat{\mathbf{J}} - \frac{3[(\hat{\mathbf{u}}^*\cdot\hat{\mathbf{J}})(\hat{\mathbf{u}}\cdot\hat{\mathbf{J}})+(\hat{\mathbf{u}}\cdot\hat{\mathbf{J}})(\hat{\mathbf{u}}^*\cdot\hat{\mathbf{J}})]-2\hat{\mathbf{J}}^2}{6(J+1)(2J+1)}\right\}, \quad (8)$$

$$V_0 = \frac{3\pi c^2\Gamma}{2\omega_0^3\Delta}I, \quad (9)$$

where c is the speed of light, $\Gamma = 0.85(3)\,\mu\mathrm{s}^{-1}$ is the resonance linewidth [2], $\omega_0 = 2\pi c/\lambda_0$, and I is the light intensity. The $\hat{J}_x^2$ coupling used to create the superposition state is achieved for a linear polarization $\hat{\mathbf{u}} = \hat{\mathbf{x}}$. We discuss below several types of experimental imperfections affecting the spin dynamics.

Finite extent of the atomic sample

We first take into account the variation of intensity and polarization over the atomic sample. We focus on the atomic sample a gaussian beam linearly polarized along x and propagating along y, of waist $w \simeq 50\,\mu\mathrm{m}$ at the atom position. Due to the beam focusing, we expect a

slight polarization ellipticity away from the optical axis $\hat{\mathbf{u}} \simeq \hat{\mathbf{x}} + \mathrm{i}\theta x/w\hat{\mathbf{y}}$, where $\theta = \lambda/(\pi w) \simeq 4\,\mathrm{mrad}$ is the beam divergence. The dynamics shown in Fig. 2 is consistent with a 1/e cloud size $\sigma = 7.3(3)\,\mu\mathrm{m}$, in agreement with the size calculated from the trap geometry and the gas temperature. Given this cloud extent, we expect r.m.s. intensity variations between atoms $\delta I/I = 6\%$ and an ellipticity typically corresponding to a Stokes parameter $s_3 = 10^{-3}$.

Quantization magnetic field
We include the effect of the applied magnetic field of amplitude $B = 18.5(3)\,\mathrm{mG}$, leading to a linear Zeeman coupling. We fit its orientation from the measured spin dynamics, consistent with an angular mismatch between the quantization field direction $\hat{\mathbf{b}}$ (of components $[0.09, -0.11, 0.98]_{x,y,z}$) and the z axis.

Imperfect spin polarization
The atomic gas is prepared in the absolute ground state $|-J\rangle_z$ under a strong magnetic field $B_z = 0.5\,\mathrm{G}$. The field is ramped to the final value $B_z = 18.5(3)\,\mathrm{mG}$ in 20 ms, during which dipole-dipole interactions lead to a slight promotion of $\simeq 3\%$ of the atoms into the state $|-J+1\rangle_z$. We take into account the spin dynamics undergone by these atoms.

Light shift correction to second-order perturbation theory
Given the small detuning from resonance, we calculate the first correction to second-order perturbation theory in the light shift. For a light field linearly polarized along x, we obtain the expression

$$\hat{V} = \hbar\omega\Big\{\hat{J}_x^2 + \frac{\omega}{\Delta}\left[(2J^2 + 3J + 1)\hat{J}_x^2 + \hat{J}_x^4\right] + \mathcal{O}[(\omega/\Delta)^2]\Big\}. \tag{10}$$

The first correcting term leads to a renormalization of the coupling frequency ω, corresponding to a $\sim 20\%$ reduction of ω for our experimental parameters. The expected (renormalized) value $\omega = 2\pi \times 1.95(10)\,\mathrm{MHz}$ agrees well with the value $\omega = 2\pi \times 1.98(1)\,\mathrm{MHz}$ fitted from the spin dynamics. The second term $\propto \hat{J}_x^4$ leads to a slight modification of the spin dynamics, but its effect remains below the experimental noise.

Light intensity response time
The light pulse shape is controlled using an acousto-optic modulator, leading to a finite response time in the 10-100 ns range. By solving numerically the spin dynamics with the actual pulse shape, we estimate that the finite response time leads to minor differences compared to square pulses of same area.

Incoherent light scattering
We model the effect of incoherent light scattering, taking into account Rayleigh and Raman scatterings using a Monte Carlo wavefunction method [3] . For the detuning chosen in our experiments, we estimate a light scattering probability of 0.7% for the light pulse duration required to produce the superposition state.

Imperfection	correction to G
Intensity inhomogeneity	-1.43
Polarization ellipticity	-0.41
B_z field amplitude	-0.22
$\hat{\mathbf{b}}$ and $\hat{\mathbf{z}}$ angle mismatch	-0.34
Imperfect initial state polarization	-0.06
$\hat{J}_x^4$ correction	-0.18
Light intensity response time	-0.18
Incoherent light scattering	-0.09
Combined correction	-1.52

Supplementary Tab. I. Balance of experimental imperfections in the reduction of the metrological gain.

We compare the effects of the different imperfections in Tab. I. For each imperfection, we calculate the resulting decrease of the metrological gain G with respect to the maximum value of $2J = 16$. The dominant imperfection stems from the finite extent of the atomic gas, leading to inhomogeneous light intensity and to polarization ellipticity. Combining all effects together, we estimate a maximum metrological gain of $G = 14.5$, consistent with the measured value $G = 13.9(1.1)$.

Supplementary Note 3: Metrological gain versus measurement scheme

We discuss in the main text two methods to probe magnetic fields using the kitten state, based on the evolution of the projection probability distributions $\Pi_m(\phi)$ along equatorial directions shown in Fig. 3a. The first method uses the oscillatory behavior of the parity $P(\phi)$ of these distributions, and the second is based on the variation of the Hellinger distance $d_{\mathrm{H}}(\phi, \phi')$ between probability distributions for different phases ϕ and ϕ'.

A similar analysis can be performed using the non-linear detection scheme, which leads to the probability distributions $\Pi_m(\phi)$ shown in Fig. 3b. We first exploit the measured magnetization oscillations shown in Fig. 3c. We evaluate the measurement precision using the general formula (9) with the observable $\hat{O} = \hat{J}_z$, leading to the expression of the metrological gain $G = 2J(A/\Delta J_z)^2$, where $A = 6.0(2)$ is the oscillation amplitude and $\Delta J_z^2 = 49(1)$ is the spin projection variance (measured for the $m_z \simeq 0$ data). A second measurement scheme consists in quantifying the variations of the probability distributions $\Pi_m(\phi)$ using the Hellinger distance, following the same procedure than for the data of Fig. 3a.

We show in Tab. II the metrological values obtained from the four measurement schemes discussed above. For both data sets we find that exploiting the variations of the Hellinger distance $d_{\mathrm{H}}(\phi, \phi')$ leads to the highest G values, both of them being compatible with the upper bound $2\Delta J_z^2/J = 14.3(1)$.

Supplementary Note 4: Dephasing due to magnetic field fluctuations

As discussed in the main text, we mainly attribute the

Spin projection on equatorial directions	G
Parity oscillation	8.8(4)
Variations of probability distributions	13.9(1.1)
**Non-linear detection**	G
Magnetization oscillation	11.2(1.3)
Variations of probability distributions	14.0(9)

Supplementary Tab. II. Metrological gain values corresponding to the measurement schemes discussed in the main text. The first scheme consists in measuring the spin projection on equatorial directions (see Fig. 3a), and using either the parity oscillations or the variations of the full probability distributions. The second scheme uses a non-linear evolution, and using the magnetization oscillations or the variations of the full probability distributions (see Fig. 3b).

observed decoherence to magnetic field fluctuations along z. The dephasing due to this classical noise can be modeled using standard techniques from nuclear magnetic resonance [4–6] . We write the magnetic field as $B_z(t) = B_z^0 + b(t)$ with $\langle b(t)\rangle = 0$. Such a field induces a noise in the Larmor rotation angle

$$\delta\phi(t) = \frac{\mu_B g_J}{\hbar}\int_0^t \mathrm{d}t' b(t'). \tag{11}$$

For a coherent state prepared on the equator, this noise leads to the decay of transverse spin $J_\perp(t) = J\left\langle e^{i\delta\phi(t)}\right\rangle$. For a superposition state $|\psi_{\text{kitten}}\rangle$ it reduces the extremal coherence as $|\rho_{-J,J}| = \left\langle e^{i2J\delta\phi(t)}\right\rangle$.

A purely Markovian evolution would be expected for white magnetic field noise, leading to phase diffusion behavior [7]. The Markovian approximation corresponds to phase diffusion without memory, corresponding to $\langle b(t)b(t')\rangle = 2D\,\delta(t-t')$ [8] . For gaussian noise statistics, we use $\left\langle e^{in\delta\phi(t)}\right\rangle = e^{-n^2\left\langle\delta\phi(t)^2\right\rangle/2}$, with a diffusive phase noise $\left\langle\delta\phi(t)^2\right\rangle = 2D(\mu_B g_J/\hbar)^2 t$, leading to exponential damping of coherences, of 1/e times $\tau_0 = (\hbar/\mu_B g_J)^2 D$ for the transverse spin $J_\perp$ and $\tau = \tau_0/(2J)^2$ for the extremal coherence $|\rho_{-J,J}|$.

In our experiment, we rather expect typical magnetic field variations to occur on timescales much larger than the $\sim$ 100 µs decoherence timescale. In this regime, the magnetic field can be considered as static during a single realization of the experiment, and decoherence arises from shot-to-shot fluctuations. We then expect $\delta\phi(t) = \mu_B g_J bt/\hbar$, which obviously leads to the relationship

$$\left\langle e^{i2J\delta\phi(t)}\right\rangle = \left\langle e^{i\delta\phi(2Jt)}\right\rangle.$$

This relationship implies that the damping of extremal quantum coherences after a duration t is equal to the damping of the coherence of coherent states after a duration $2Jt$, consistently with our observations.

Since each atom carries a magnetic moment $\sim 10\,\mu_B$, we also expect an additional magnetic field created by the atomic sample itself, but we estimate its contribution to the coherence damping rate to be one order of magnitude smaller than external magnetic field effects.

Supplementary references

[1] Kitagawa, M. & Ueda, M. Squeezed spin states. *Phys. Rev. A* **47**, 5138–5143 (1993).

[2] Gustavsson, M., Lundberg, H., Nilsson, L. & Svanberg, S. Lifetime measurements for excited states of rare-earth atoms using pulse modulation of a cw dye-laser beam. *JOSA* **69**, 984–992 (1979).

[3] Dalibard, J., Castin, Y. & Mølmer, K. Wave-function approach to dissipative processes in quantum optics. *Phys. Rev. Lett.* **68**, 580 (1992).

[4] Allen, L. & Eberly, J. H. *Optical resonance and two-level atoms*, vol. 28 (Courier Corporation, 1975).

[5] Luczka, J. Spin in contact with thermostat: Exact reduced dynamics. *Physica A* **167**, 919–934 (1990).

[6] Palma, G. M., Suominen, K.-A. & Ekert, A. K. Quantum computers and dissipation. In *Proc. R. Soc. Lond. A*, vol. 452, 567–584 (The Royal Society, 1996).

[7] Ferrini, G., Spehner, D., Minguzzi, A. & Hekking, F. W. Noise in Bose Josephson junctions: Decoherence and phase relaxation. *Phys. Rev. A* **82**, 033621 (2010).

[8] Van Kampen, N. G. *Stochastic processes in physics and chemistry*, vol. 1 (Elsevier, 1992).
